# Reliability-Based Design Analysis for FRP Reinforced Compression Yield Beams

**DOI:** 10.3390/polym14224846

**Published:** 2022-11-11

**Authors:** Lin Feng, Peng-Da Li, Xiao-Xu Huang, Yu-Fei Wu

**Affiliations:** 1 College of Civil and Transportation Engineering, Shenzhen University, Shenzhen 518060, China; 2 Guangdong Provincial Key Laboratory of Durability for Marine Civil Engineering, Shenzhen University, Shenzhen 518060, China; 3 School of Engineering, RMIT University, 376-392 Swanston St, Melbourne, VIC 3001, Australia

**Keywords:** reliability analysis, compression yielding, beams, ductility, FRP-reinforced concrete, Monte Carlo simulation

## Abstract

Fiber-reinforced polymers (FRPs) provide promising prospects for replacing steel bars in traditional reinforced concrete structures. However, the use of FRP as tension bars in concrete beams leads to insufficient ductility because of its elastic characteristics. A newly developed compression-yielding (CY) beam has successfully solved this issue. Instead of tensile reinforcement yield, the ductile deformation of a CY beam is realized by the compression yield of a CY block in the compressive region. Another important feature is that the CY block is also the fuse of the beam, where material damage to the beam is concentrated in the CY block region and can be easily replaced. As a load-bearing recoverable and ductile structure, it is necessary to conduct a reliability-based design analysis and recommend reduction factors for this new structure. In this study, the function for calculating the failure probability of CY beams is proposed, semi-probabilistic design recommendations are presented, and Monte Carlo simulation (MCS) is adopted as a reliability analysis method. This study discusses the influence of the possible characteristics of the critical variables on reliability and provides the reliability index with different reduction factors to guide the design of the CY beam. These analyses indicate that the reliability index can be improved from the material design of the CY block in greater strength *f_b_*, smaller depth, smaller coefficient of variation of *f_b_*, and yield modulus ratio *ξ*. This study also shows that compared with the design of FRP concrete beams, the ductile failure mode of the CY beams allows a lower safety factor to meet safety requirements, which significantly reduces construction costs and avoids over-designing the load-bearing capacity.

## 1. Introduction

The durability and service life of reinforced concrete (RC) structures, to a large extent, must consider rebar corrosion, especially in marine environments [1]. To solve the problem of structural deterioration caused by steel corrosion, fiber-reinforced polymers (FRPs) offer promising prospects by replacing steel reinforcement in concrete structures [2]. However, the characteristics of the linear elasticity of FRP materials lead to insufficient ductility in FRP-RC elements design [3]. Compared with ductile structures, brittle structures require a smaller probability of failure [4,5]. For FRP-RC structures, a larger safety margin is recommended by ACI 440 [5,6], which means that the loading capacity is substantially underestimated. The over-design method inevitably results in significant waste in construction costs and is unrealistic in large structures [7]. As Naaman commented, “Unless ductility requirements are satisfied, FRP materials cannot be used reliably in structural engineering applications” [7]. In previous investigations, many methods to improve the structural ductility of FRP-RC members have been proposed. For example, prestressed FRP bars can be combined with steel reinforcement to achieve ductility [8]; the FRP beam can be designed as an over-reinforced member, which increases the ductility of concrete by providing restraint [9]. Compared with normal concrete, geopolymer concrete reinforced with glass fiber reinforced polymer (GFRP) [10] and high-strength concrete reinforced with hybrid-GFRP [11] exhibited a higher ductility of the beams. The extensive explorations mentioned above show that the brittle damage of universal FRP-RC structures is unavoidable unless the mechanism of the structure is changed.

**Figure 1:**
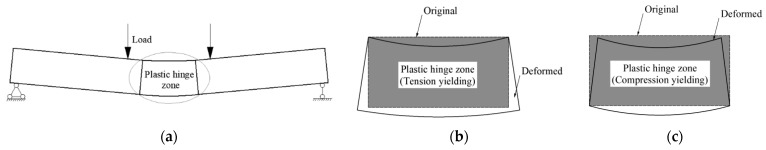
Plastic hinge mechanism [10]. (**a**) Plastic hinge zone in the flexural structure, (**b**) conventional RC member, (**c**) CY member.

**Figure 2:**
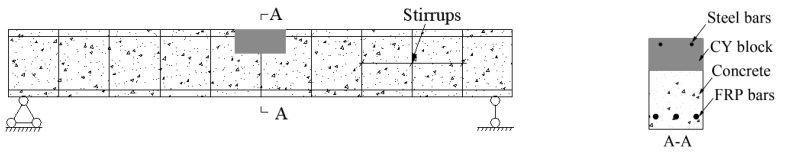
FRP-RC beam with a CY block [11].

Usually, the ductile deformation is predominantly concentrated in the plastic hinge zone, which is from the rebar’s plastic deformation (Figure 1a). However, the ductility of FRP-RC structures cannot be derived from the tensile reinforcement yield (Figure 1b). Based on a fundamental understanding of plastic deformation, a new concept named compression yielding was proposed by Wu et al. [12] (Figure 1c), which can effectively meet the ductility requirement of FRP-RC beams. The ductility of the structure can be achieved by a special component with adequate strength and ductility in the compression area [13], i.e., CY block (Figure 2). When an overload occurs, the CY block will force the structure to deform in a plastic manner to avoid the abrupt rupturing of the FRP bar or the crushing of concrete. Furthermore, the CY block can be replaced after a certain deformation so that the structure can work functionally again, in which the CY block acts as a fuse in the structural system [14]. The CY beam has the following benefits: (1) It avoids excessive waste of the loading capacity of the FRP structure by transforming a dangerous failure mode (brittle failure) into a safer one (ductile failure). (2) The initial bearing capacity of the beam can be restored by replacing the CY block.

Up to now, many breakthroughs have been made in the research of CY beams. Traditional engineering materials cannot meet the special properties required for a CY block, which is sufficiently rigid under service load while highly ductile movement occurs. After extensive exploration, two kinds of CY blocks have been developed to realize special performance: (1) mild steel blocks with holes [14,15] and (2) slurry-infiltrated fiber concrete (SIFCON) blocks with holes [16,17]. Numerical simulation and experimental results have shown that the CY blocks could significantly improve the ductility of FRP-RC beams [15,18]. Apart from this, a performance-based design method and a program for designing the CY beams have been proposed, which theoretically validated that the CY approach can solve the ductility problem and further promoted the application of this new technique [19,20].

To sum up, the CY beam has the characteristics of ductility and recoverable bearing capacity. Thus, the reduction factor adopted in the design process for FRP-RC structures is not appropriate for the new CY structures. In addition, the utilization of a CY block unavoidably involves more uncertainties in the beam design, which further affects structural safety. In this study, the function for calculating the failure probability of the CY beam is proposed, and the reliability-based design analysis is performed to identify the safety level of the structure and give a recommendation regarding the design factors. The level of structural safety is measured by the reliability index [21]. The reliability index of the structure can be calculated based on the load and resistance factor design (LRFD) principle [21], which is:

(1) $$\phi R_{n}\left(f_{ck},f_{bk},\ldots \right)\geq \sum^{\;}\gamma_{i}Q_{i}$$

where *R_n_* is the nominal resistance with a resistance reduction factor of *ϕ*; *f_ck_* and *f_bk_* are the characteristic strengths of the concrete and CY block, respectively; and *Q_i_* is the load effect with the corresponding partial safety factor of *γ_i_*.

This paper is organized into the following sections. In Section 2, the design methods for CY beams and FRP-RC beams are introduced. In Section 3, the statistical characteristics of design variables are described, including the model error, geometry, materials, and loads. In Section 4 and Section 5, the results of the reliability analysis of the strength ultimate limit state and deformation ultimate limit state are calculated, respectively. In Section 6, the concept of CY beam failure is presented, and finally, the reliability index of CY beams with different reduction factors is obtained.

## 2. Design Procedure for CY Beams and FRP-RC Beams

### 2.1. Compression Yielding FRP-RC Beams

#### 2.1.1. Three Types of Moment–Curvature Responses

**Figure 3:**
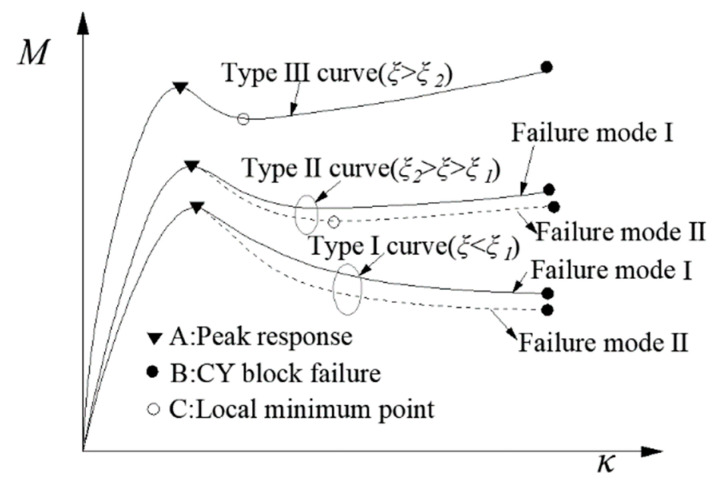
Moment–curvature responses.

**Figure 4:**
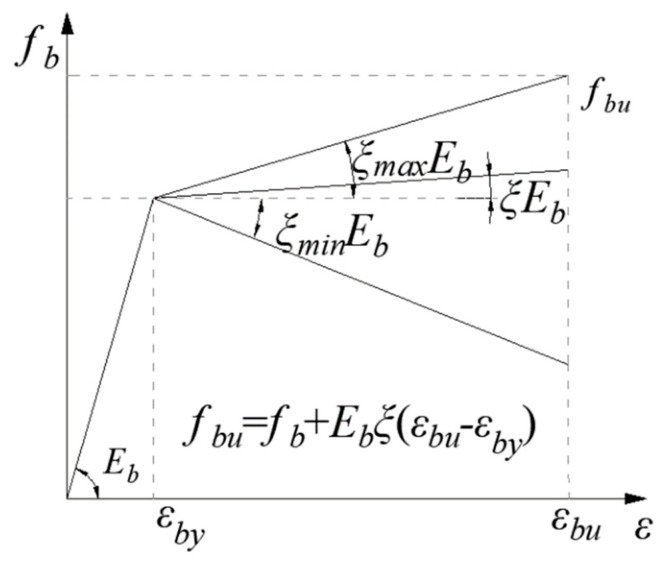
Stress–strain relationships for the CY block.

The derivation of the design equation for a CY beam was according to the minimum cross-sectional area of the CY block (Figure 2). The three typical moment–curvature responses at the CY zone are shown in Figure 3, the stress–strain relationships for the CY block is shown in Figure 4. The post-yield modulus ratio *ξ* of the CY block determines the type of the curves [17]: (1) when *ξ* ≤ *ξ*_1_ (Type I), the response curve rises monotonically before reaching the peak value (point A) and then decreases; (2) when *ξ*_1_≤ *ξ* ≤*ξ*_2_ (Type II) after point A, the response curve exhibits an initial decline until the minimum value *M_lm_* (point C) is reached. The moment resistance at the failure point of the CY block (point B) is smaller than point A; (3) when *ξ* > *ξ*_2_ (Type III), the value of point B is larger than that of point A. The boundary value of the ratios *ξ*_1_ and *ξ*_2_ are obtained by solving the formula *M_m_* = *M_u_* and *κ_lm_ = κ_u_*, respectively, where *M_m_* is the moment at point A, *κ_lm_* is the curvature of point C, and *M_u_* and *κ_u_* are the moments and curvature at point B, respectively.

To avoid concrete crushing, the compressive strength of concrete *f_c_* must be larger than the peak strength of the CY block *f_bu_* and the value of *f_bu_* must be positive, which also provides a range of *ξ*:

(2) $$\xi \leq \xi_{max}=\frac{f_{c}-f_{b}}{\left(\varepsilon_{bu}-\varepsilon_{by}\right)E_{b}}$$

(3) $$\xi \geq \xi_{min}=-\frac{\varepsilon_{by}}{\left(\varepsilon_{bu}-\varepsilon_{by}\right)}$$

#### 2.1.2. Three Cases and Their Corresponding Peak Responses

**Figure 5:**
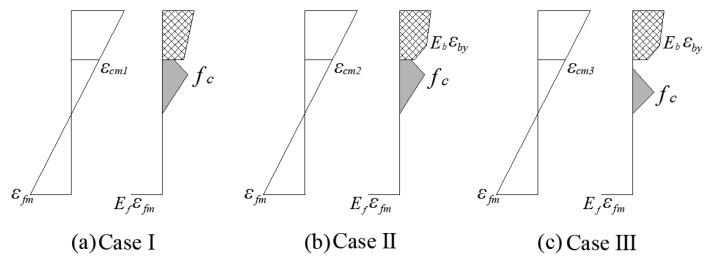
Strain distributions of the cross-section in the CY zone at the peak point A.

This study considered the peak moment *M_m_* at point A to derive the ultimate limit state functions. There are three possible cross-section strain distributions at peak point A for each type of moment–curvature response (Figure 5), named Cases I, II, and III. The strains at the concrete–CY block junction *ε_cmi_* (where the subscript *i* = 1, 2, 3 indicates cases I, II, III, respectively) are listed below.

For Case I (Figure 5a), the CY block yields completely at the onset of *M_m_*, which gives:

(4) $$\varepsilon_{cm1}=\sqrt{\left(\varepsilon_{fm}^{2}+2\varepsilon_{cu}\varepsilon_{fm}+\varepsilon_{cu}^{2}/n\right)/S_{1}}-\varepsilon_{fm}$$

where

(5) $$S_{1}=1-\frac{\left(n-1\right)\left(2-\eta \right)\eta \xi E_{b}\varepsilon_{cu}}{n{\left(1-\eta \right)}^{2}f_{c}}>0$$

where *ξ* and *ε_by_* satisfy the following two inequalities:

(6) $$\xi \leq \frac{{\left(1-\eta \right)}^{2}f_{c}\varepsilon_{cu}}{\left(2-\eta \right)\eta E_{b}{\left(\varepsilon_{fm}+\varepsilon_{cu}\right)}^{2}}$$

(7) $$\varepsilon_{by}+\varepsilon_{fm}-\sqrt{\left(\varepsilon_{fm}^{2}+2\varepsilon_{cu}\varepsilon_{fm}+\varepsilon_{cu}^{2}/n\right)/S_{1}}\leq 0$$

For Case II (Figure 5b), the CY block has not completely failed when *M_m_* is reached, and the concrete at the interface has not reached the ultimate strain. In this case, *ε_cm_*_2_ is provided as follows:

(8) $$\varepsilon_{cm2}=\sqrt{\frac{f_{c}{\left(\varepsilon_{fm}+\varepsilon_{cu}\right)}^{2}/\left(\varepsilon_{cu}-\varepsilon_{0}\right)+\left(1-\xi \right)E_{b}{\left(\varepsilon_{fm}+\varepsilon_{by}\right)}^{2}-f_{c}\varepsilon_{cu}}{f_{c}/\left(\varepsilon_{cu}-\varepsilon_{0}\right)+E_{b}-\xi E_{b}/{\left(1-\eta \right)}^{2}}}-\varepsilon_{fm}$$

where

(9) $$\varepsilon_{by}+\varepsilon_{fm}-\sqrt{\frac{f_{c}\varepsilon_{cu}}{\left(1-\xi \right)E_{b}}+\left[1-\frac{\xi }{{\left(1-\eta \right)}^{2}}\right]\frac{{\left(\varepsilon_{fm}+\varepsilon_{cu}\right)}^{2}}{1-\xi }}⩽0$$

(10) $$\varepsilon_{by}+\varepsilon_{fm}-\sqrt{\left(\varepsilon_{fm}^{2}+2\varepsilon_{cu}\varepsilon_{fm}+\varepsilon_{cu}^{2}/n\right)/S_{1}}⩾0$$

For Case III (Figure 5c), the concrete at the interface has completely crushed but the CY block has not completely yielded at the onset of *M_m_*. In this case, *ε_cm_*_3_ is given as follows:

(11) $$\varepsilon_{cm3}=\sqrt{\frac{\left(1-\xi \right){\left(\varepsilon_{fm}+\varepsilon_{cu}\right)}^{2}}{1-\xi /{\left(1-\eta \right)}^{2}}-\frac{f_{c}\varepsilon_{cu}}{\left[1-\xi /{\left(1-\eta \right)}^{2}\right]E_{b}}}-\varepsilon_{fm}$$

where

(12) $$\varepsilon_{by}+\varepsilon_{fm}-\sqrt{\frac{f_{c}\varepsilon_{cu}}{\left(1-\xi \right)E_{b}}+\left[1-\frac{\xi }{{\left(1-\eta \right)}^{2}}\right]\frac{{\left(\varepsilon_{fm}+\varepsilon_{cu}\right)}^{2}}{1-\xi }}⩾0$$

(13) $$\varepsilon_{by}+\varepsilon_{fm}-\sqrt{\frac{f_{c}\varepsilon_{cu}{\left(1-\eta \right)}^{2}}{\eta \left(1-\eta \right)\xi E_{b}}}<0$$

Based on Equations (2)–(13), at the onset of *M_m_*, the FRP tension force (*F_f_*) and the curvature *κ_m_* of the CY beam are provided as follows:

(14) $$F_{f}=F_{i}=\left(1+\beta_{1i}+\beta_{2i}\right)E_{b}\varepsilon_{by}\eta bd+\frac{b\varepsilon_{cu}f_{c}}{2\kappa_{emi}}+E_{s}\varepsilon_{sy}\rho_{s}bd$$

(15) $$\kappa_{m}=\kappa_{mi}=\frac{\varepsilon_{fm}+\varepsilon_{cmi}}{\left(1-\eta \right)d}$$

where

(16) $$\kappa_{emi}=\frac{\kappa_{mi}}{1-\frac{n}{n-1}{\left(1-\frac{\varepsilon_{\mathrm{cmi}}}{\varepsilon_{\mathrm{cu}}}\right)}^{2}},\kappa_{em3}=\kappa_{m3}$$

(17) $$\beta_{11}=\frac{\left(\varepsilon_{cm1}-\varepsilon_{by}\right)\xi }{\varepsilon_{by}},\left(\mathrm{for}\;\mathrm{Case}\;i=1\right),\;\beta_{1i}=-\frac{{\left(\varepsilon_{by}-\varepsilon_{cmi}\right)}^{2}}{2\eta d\varepsilon_{by}\kappa_{mi}},(\mathrm{for}\;\mathrm{Case}\;i=2,\;3)$$

(18) $$\beta_{21}=\frac{\kappa_{m1}\eta d\xi }{2\varepsilon_{by}},\left(\mathrm{for}\;\mathrm{Case}\;i=1\right),\;\beta_{2i}=\frac{{\left(\kappa_{mi}\eta d+\varepsilon_{cmi}-\varepsilon_{by}\right)}^{2}\xi }{2\eta d\varepsilon_{by}\kappa_{mi}},(\mathrm{for}\;\mathrm{Case}\;i=2,\;3)$$

Overall, the peak moment (point A in Figure 3) *M_m_* is given by:

(19) $$M_{m}=E_{b}\varepsilon_{by}\eta bdZ_{bi}+\frac{b\varepsilon_{cu}f_{c}}{2\kappa_{emi}}Z_{ci}+E_{s}\varepsilon_{sy}\rho_{s}\left(1-\zeta \right)bd^{2}$$

where

(20) $$Z_{b1}=\left(1+\beta_{11}\right)\left(1-\eta /2\right)d+\beta_{21}\left(1-\eta /3\right)d,\;\left(\mathrm{for}\;\mathrm{Case}\;i=1\right)$$

(21) $$Z_{bi}=\left(1-\eta /2\right)d+\beta_{1i}\left[\left(1-\eta \right)d+\frac{\varepsilon_{by}-\varepsilon_{cmi}}{3\kappa_{mi}}\right]+\beta_{2i}\left[d-\frac{\kappa_{mi}\eta d+\varepsilon_{cmi}-\varepsilon_{by}}{3\kappa_{mi}}\right],\;\left(\mathrm{for}\;\mathrm{Case}\;i=2,\;3\right)\;$$

(22) $$Z_{ci}=\left[1-\frac{3n\varepsilon_{0}\varepsilon_{cmi}^{2}-\varepsilon_{cmi}^{3}-3n\varepsilon_{0}^{2}\varepsilon_{cmi}+n\varepsilon_{0}^{3}}{\left(\varepsilon_{fm}+\varepsilon_{cmi}\right)\left(6n\varepsilon_{cmi}\varepsilon_{0}-3\varepsilon_{cmi}^{2}-3n\varepsilon_{0}^{2}\right)}\right]\left(1-\eta \right)d,\;\left(\mathrm{for}\;\mathrm{Case}\;i=1,\;2\right)$$

(23) $$Z_{c3}=\frac{3n\varepsilon_{fn}+\left(n+1\right)\varepsilon_{cu}}{3n\left(\varepsilon_{fm}+\varepsilon_{cm3}\right)}\left(1-\eta \right)d\;\left(\mathrm{for}\;\mathrm{Case}\;i=3\right)$$

In addition, there are two types of failure modes defined for the CY beam: (1) The ultimate strain *ε_bu_* of the CY block has been reached. (2) After reaching the peak point (A), the moment resistance drops below the allowable rate of moment drop *δ_d_* (Equation (24)) before CY block failure.

(24) $$\delta_{d}=\frac{M_{m}-M_{\min}}{M_{m}}$$

where *M_min_* is the minimum value of the resistance, which equals *M_lm_* (at point C in type II and type III curves in Figure 3) or *M_u_* (at point B of the type I curve in Figure 3).

**Figure 6:**
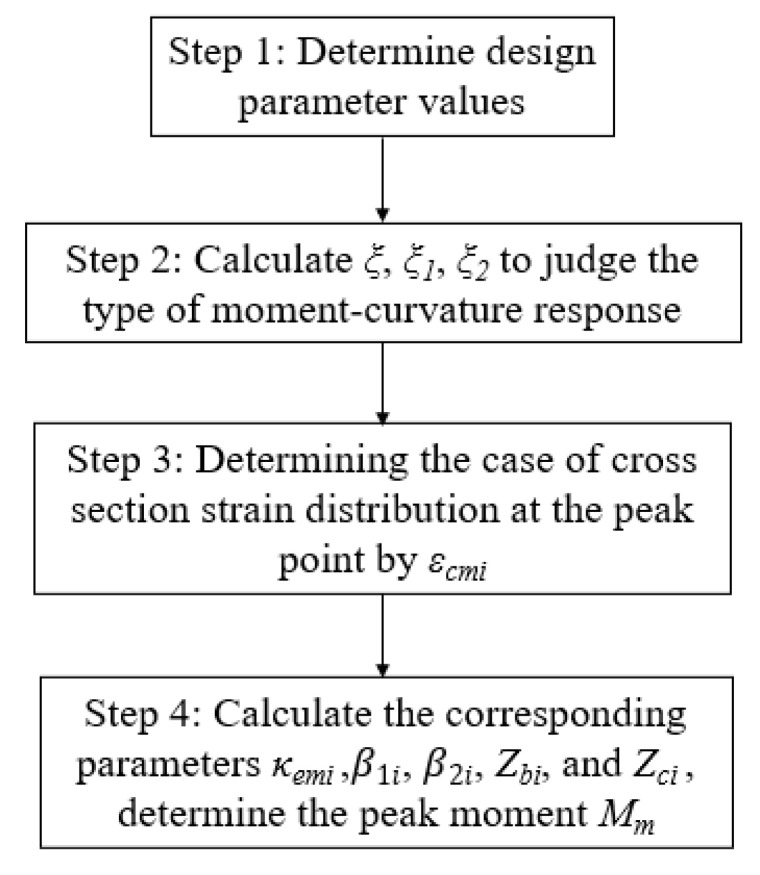
Flowchart of the design procedure for CY beams.

The flowchart of the design procedure for CY beams is shown in Figure 6.

### 2.2. Design Procedure for FRP-RC Beams According to ACI-440

In the ACI 440 guideline, the actual value of the balanced FRP ratio, *ρ_fb_* determines the failure mode of the FRP-RC beam, which is given by:

(25) $$\rho_{fb}=0.85\beta_{1}\frac{f_{c}^{\prime }}{f_{fu}}\frac{E_{f}\varepsilon_{cu}}{E_{f}\varepsilon_{cu}+f_{fu}}$$

The formulation is governed by the failure mode and is used to compute the beam resistance. For the case of *ρ_f_* > *ρ_fb_*, concrete crushing occurs. The FRP ratio *ρ_f_* is defined by *A_f/_*(*bd*), where *A_f_* is the cross-section area of the FRP bar. The bending resistance, *M_n_*, is provided by:

(26) $$M_{n}=A_{f}f_{f}\left(d-\frac{a}{2}\right)$$

where

(27) $$a=\frac{A_{f}f_{f}}{0.85f_{c}^{\prime }b}$$

(28) $$f_{f}=\sqrt{\frac{{\left(E_{f}\varepsilon_{\mathrm{cu}}\right)}^{2}}{4}+\frac{0.85\beta_{1}f_{c}^{\prime }}{\rho_{f}}E_{f}\varepsilon_{\mathrm{cu}}}-0.5E_{f}\varepsilon_{\mathrm{cu}}$$

In Equation (26), the tensile stress of the FRP reinforcement *f_f_* is limited to the designed FRP tensile strength, *f_fu_*. For the case of *ρ_f_* < *ρ_fb_*, FRP rupture occurs and the *M_n_* is given by:

(29) $$M_{n}=0.8A_{f}f_{fu}\left(d-\frac{\beta_{1}}{2}\left(\frac{\varepsilon_{cu}}{\varepsilon_{cu}+\varepsilon_{fu}}\right)d\right)$$

The strength design concept implies that the demand bending moment *M_f_* (calculated from the factored loads) must be satisfied the nominal bending strength *M_n_*, which gives:

(30) $$\phi M_{n}\geq M_{f}$$

The resistance reduction factors *ϕ* are given by:

(31) $$\phi =\{\begin{array}{ccc} 0.5 & \mathrm{for} & \rho_{f}\leq \rho_{fb}\\ 0.3+0.25\frac{\rho_{f}}{\rho_{fb}} & \mathrm{for} & \rho_{fb}\leq \rho_{f}\leq 1.4\rho_{fb}\\ 0.65 & \mathrm{for} & \rho_{f}\geq 1.4\rho_{fb} \end{array}$$

### 2.3. Comparison of Design Concepts

The failure modes determine the different design concepts for FRP-RC beams and CY beams. FRP-RC beams do not yield in a brittle failure mode, which is caused by the rupture of the tensile FRP reinforcement or concrete crushing. Thus, the method recommended by ACI 440 adopts a large safety margin, with 65% of the nominal bending resistance of the structure due to concrete crushing or 50% of that for FRP rupture (Equation (31)) [6]. For CY beams, however, this kind of brittle failure mode can be avoided because the total compressive strength is designed to be smaller than the tensile strength of the beams, and hence, the FRP will not reach its tensile capacity throughout the bending process.

The incorporation of CY blocks produces the following effects: (1) Brittle failure is transformed to a safer failure mode. The CY block acts as a fuse, and the large ductile and visible deformation of the structure prevents further overloading, thus reducing the probability of failure. (2) The repair of members is simple and fast by replacing the fuse (i.e., CY block) rather than the entire beam, which will greatly reduce the maintenance cost and duration. Therefore, for CY beams, the target reliability in the design should be referred to as a ductile structure and the resistance reduction factor given by Equation (31) for FRP-RC beam should not be used. The following section is a detailed reliability-based analysis for the CY beam.

## 3. Statistical Characteristics of the Design Variables

Reliability analysis requires supporting the database and probability models of variables to characterize the corresponding uncertainties. The uncertainty in the reliability analysis of CY beams mainly comes from four parts, i.e., geometries, material properties, load, and calculation model. Defining the statistical characteristics of these variables is a crucial step for reliability-based design analysis for CY structures, which is listed in this section.

### 3.1. Model Uncertainty

The model uncertainty factor *K_p_* in the resistance analysis is mainly caused by approximations and assumptions, which can be treated as a random variable, given by:

(32) $$K_{p}=M_{exp}/M_{pre}$$

where *M_exp_* and *M_pre_* denote the experimental values and predicted values, respectively.

Although the experimental work confirms the validity of the CY mechanism [14,16], the existing experimental data are insufficient for the analysis of *K_p_* due to the large and difficult beam-related experiments. Similar to the FRP-RC beam, however, the establishment of the loading capacity model for the CY beam is also based on the semi-empirical method that employs the following assumptions: (1) the plane sections remain plane; (2) the tensile strength of the concrete is neglected; and (3) the FRP is linearly elastic. On the other hand, this paper involves comparing two structures, so the same *K_p_* as that of FRP-RC is used for CY beams. The *K_p_* of CY beams will be updated when the experimental work being performed by the research group is completed. Table 1 shows the *K_p_* of *M_n_* recommended by ACI 440. Especially the failure type of FRP-RC beams in Table 1 is compression damage (concrete crushing), which is consistent with the CY beams (over-reinforced).

**Figure 7:**
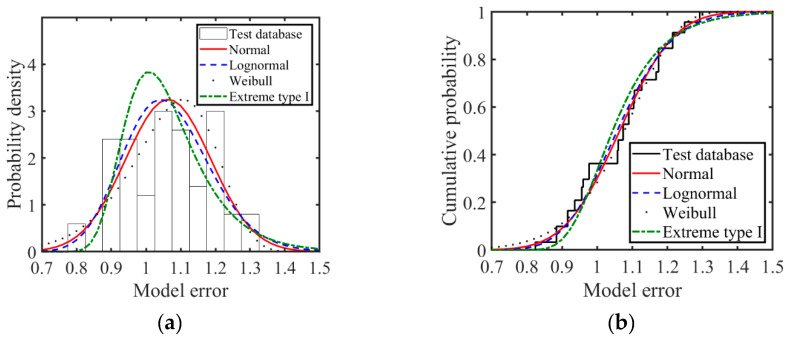
K-S testing of the distribution of model uncertainty. (**a**) PDF, (**b**) CDF.

To determine the statistical characteristics of *K_p_*, the Kolmogorov–Smirnov (K-S) test was performed by assuming several possible probability distributions with a significance level of 0.05 (i.e., Normal, Lognormal, Weibull, and Extreme type I distribution). Figure 7 shows the fitting results of the probability density function (PDF) and cumulative probability function (CDF) of *K_p_* with the hypothetical probability distribution, and Table 2 presents the K-S result value of *K_p_*. As illustrated in Table 2, the value of “1” in the first column indicates that the hypothesis is rejected and “0” means it is acceptable. Additionally, the higher *p* value in the second column indicates that the corresponding assumption is closer to the real one. The fitting results of the normal distribution are in good agreement with the *K_p_* distribution, with a mean value of 1.06 and a standard deviation of 0.12.

### 3.2. Uncertainties of the Resistance Design Variables

In this work, the statistical characteristics of the materials and geometric dimensions adopted are based on previous research, as shown in Table 3. The statistical data for CY material is based on the experimental results of the perforated SIFCON reported in [17].

### 3.3. Load Uncertainties

Considering the most dominant combination of one live load (*L*) and one dead load (*D*), the design load *S_d_* is expressed as follows:

(33) $$S_{d}=\gamma_{D}D_{n}+\gamma_{L}L_{n}$$

where *D_n_* and *L_n_* are the nominal dead and nominal live load, respectively. *γ_D_* and *γ_L_* are the partial safety factors for the dead load and live load, respectively. In LRFD for buildings [45], *γ_D_* and *γ_L_* are commonly defined as 1.2 and 1.6, respectively. The statistical data of the load are listed in Table 3.

## 4. Reliability Analysis of CY Beams Based on the Ultimate Flexural Strength

### 4.1. Limit State Function of the Ultimate Flexural Strength

Reliability analysis is a discipline that seeks to quantify the level of safety of a system. The estimations of the probability of failure (*P_A_*) for the designed CY beams with respect to the ultimate flexural strength can be obtained based on the following limit state function (LSF) *Z_A_*:

(34) $$Z_{A}=K_{p}M-D-L$$

where *K_p_* is the model uncertainty factor (Section 3.1), and *M* is the resistance-predicted value (Equation (19)), the random resistance *R* can be expressed as *K_p_M*, i.e., *R* = *K_p_M*. Mean values of the design variables were applied to calculate of the mean resistance *M*. *D* and *L* are the random load effect caused by dead load and live load, respectively. The uncertainties associated with load statistics are shown in Table 3. The concept of limit state design can be expressed by

(35) $$R_{d}=S_{d}$$

where *R_d_* is the design resistance of CY beams and is expressed as follows:

(36) $$R_{d}=\phi_{A}\times M_{n}$$

where *ϕ_A_* is the reduction factor. The nominal values of the design variables were applied to calculate the nominal resistance *M_n_* (Equation (19)). Substitute Equation (33) into Equation (35), and the values *D_n_* and *L_n_* can be calculated by

(37) $$D_{n}=R_{d}/\left(\gamma_{D}+\gamma_{L}\alpha \right),\;L_{n}=\alpha R_{d}/\left(\gamma_{D}+\gamma_{L}\alpha \right)$$

where *α* is the nominal live-to-dead load ratio *L_n_*/*D_n_*.

### 4.2. Design Space

In Section 3, the random features of the adopted variables are determined, including the distribution, bias factor, and COV. Additionally, a large design space with respect to these variables is selected for reliability analysis to consider a comprehensive design situation, which covers all possible design patterns for CY beams (Case I to III in Section 2.1.2), as given in Table 4. Furthermore, to analyze the effect of the resistance reduction factor on the reliability index, seven values of *ϕ_A_* ranging from 0.55 to 0.85 with an interval of 0.05 are considered. Five live-to-dead load ratios (*α*) ranging from 0.5 to 2.5 with an interval of 0.5 are also adopted for the reliability study. In total, the variable combination in the entire design space is 3 × 3 × 3 × 5 × 4 × 5 × 7 = 18,900.

### 4.3. Reliability Analysis Method

According to probability theory, failure occurs once the value of the limit state function is less than 0, i.e., *Z_A_* < 0. Thus, the corresponding failure probability (*P_f_*) is given by:

(38) $$P_{f}=P(Z_{A}<0)$$

Another item widely used to quantify the safety level is the reliability index *β*, which represents the shortest distance between the origin point in standard normal space and the limit state function. The relationship between failure probability *P_f_* and reliability index *β* can be expressed as:

(39) $$\beta =\Phi^{-1}\left(1-P_{f}\right)$$

where $\Phi^{-1}$ is the inverse standard normal distribution.

If the PDF of *Z* (i.e., *f_z_* (*z_A_*)) is known, then *P_f_* is rewritten by:

(40) $$P_{f}=P(Z_{A}<0)=\int_{-\infty }^{0}f_{z}\left(z_{A}\right)d_{z}$$

However, the resistance *R* may be high-dimensional and *f_z_*(*z_A_*) is difficult to obtain analytically. Approximation methods and simulation methods are widely used to evaluate the probability of failure. As a representative approximation method, the principle of the first-order reliability method (FORM) [46,47] is to simplify the high-dimensional and nonlinear limit state function by performing a linear Taylor expansion and then calculating the reliability index *β*.

As a kind of simulation method, Monte Carlo simulation (MCS) [46,47] generates abundant random samples for each random variable, and the common sampling techniques includes direct sampling (DS) and importance sampling (IS). Direct sampling estimates *P_f_* by sampling according to the distribution of random variables, without other processing, see Equation (41) as follows:

(41) $$P_{f}=\frac{1}{N}\overset{N}{\underset{j=1}{\sum }}I\left[Z_{A}\left(X^{j}\right)\right]$$

where *N* is the number of random samples, and *I*[*Z_A_*(*X*)] is the indicator function with a value of 1 if *Z_A_*(*X*(*^i^*)) < 0 or 0 if *Z_A_*(*X*(*^i^*)) ≥ 0. DS is time-consuming with low accuracy for small probability events [48]. To solve this issue, IS [46,47] uses a new probability distribution to change the location of the sampling center to sample random variables (Equation (42)):

(42) $$P_{f}=\frac{1}{N}\overset{N}{\underset{j=1}{\sum }}\frac{I\left[Z_{A}\left(X^{j}\right)\right]f\left(X^{j}\right)}{p\left(X^{j}\right)}$$

where *p*(*x*) is the constructed PDF and is often set to a normal distribution with a standard deviation of the original PDF *f*(*X*). The mean value for *p*(*X*) falls on the most likely point X^∗^ calculated by FORM. Compared with MCS, the indicator function’s weight (i.e., $f\left(X^{j}\right)/p\left(X^{j}\right)$) in Equation (42) is no longer 1.

### 4.4. Reliability Analysis Procedure

**Figure 8:**
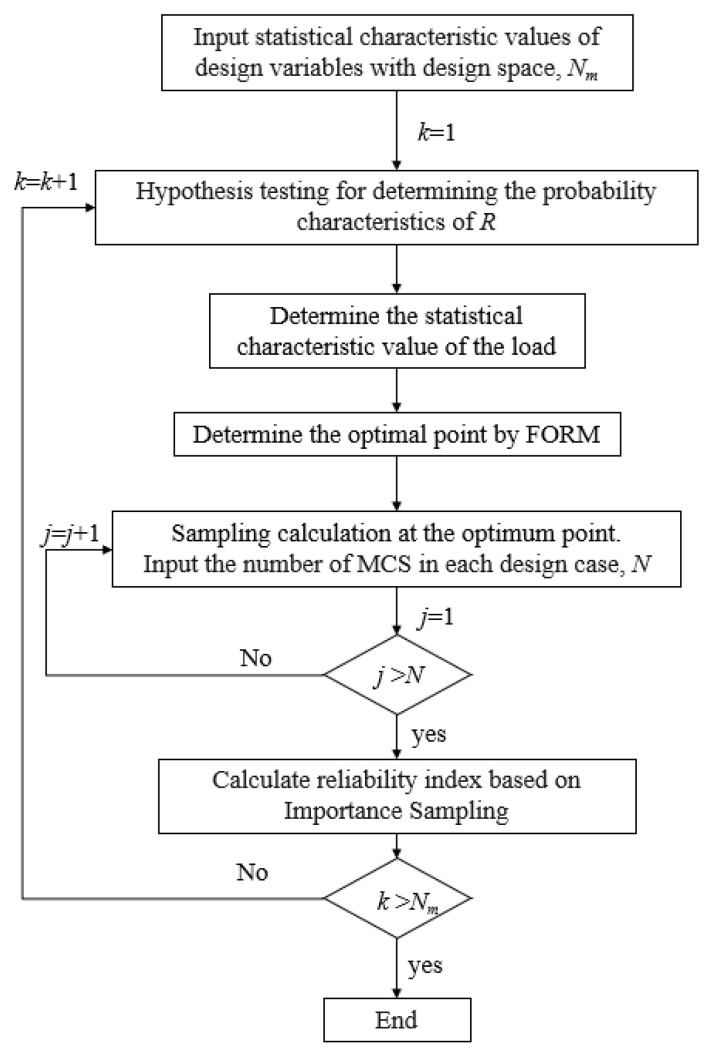
Flowchart of the reliability analysis based on IS.

In this study, IS is employed to calculate the reliability level of the CY beam. The detailed rationale for this choice is illustrated in Section 5.1 by comparing the results of the three methods (i.e., FORM, DS, and IS). There are 540 combinations of resistance, i.e., *N_m_* = 540. This paper uses the IS method to analyze 100,000 random numbers of variables, i.e., *N* = 100,000. The IS procedure is shown in Figure 8.

## 5. Results of the Reliability Analysis Based on the Ultimate Flexural Strength

To investigate the sensitivity of computation methods and design variables to the reliability index, the comparison of different reliability methods is discussed in Section 5.1. Furthermore, the average reliability index versus reliability parameters, including model uncertainty factors, geometric dimension, material properties, and load ratios, are discussed separately in Section 5.2.

### 5.1. Comparison of Different Reliability Methods

**Figure 9:**
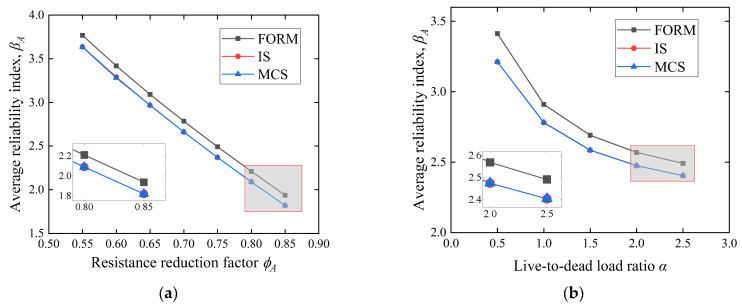
Reliability index comparison of different methods. (**a**) Effect of *ϕ_A_* on *β_A_* with different methods, (**b**) effect of *α* on *β_A_* with different methods.

To determine the accuracy and efficiency of IS, the average reliability results of the three methods (FORM, direct MCS, and IS) with *ϕ_A_* of 0.55:0.05:0.85 and *α* of 0.5:0.5:2.5 are compared, as shown in Figure 9. The result of direct MCS is close to that of IS, while the reliability index calculated by FORM is larger than the other two methods. For example, when *ϕ_A_* = 0.8, the reliability index *β_A_* calculated by FORM is 2.21, while *β_A_* evaluated by IS is 2.09. This relatively larger error is attributed to the linear Taylor expansion adopted in the FORM. In contrast, MCS is a relatively stable method; however, for small *ϕ_A_*, direct sampling may lose effectiveness for small probability events [46,47]. Therefore, it is reasonable to use the IS method in this study for efficient and accurate reliability analysis.

### 5.2. The Influence of Design Variables on the Reliability Index

The effects of each parameter on the average reliability index are discussed in this section. Notably, in Section 5.2.1, Section 5.2.2 and Section 5.2.3, the post-yield modulus *ξ* is fixed with a value of 0, while in Section 5.2.4, *ξ* is assumed to be a uniformly distributed variable between *ξ_max_* and *ξ_min_* (Equations (2) and (3)).

#### 5.2.1. Influence of Material and Geometry

**Figure 10:**
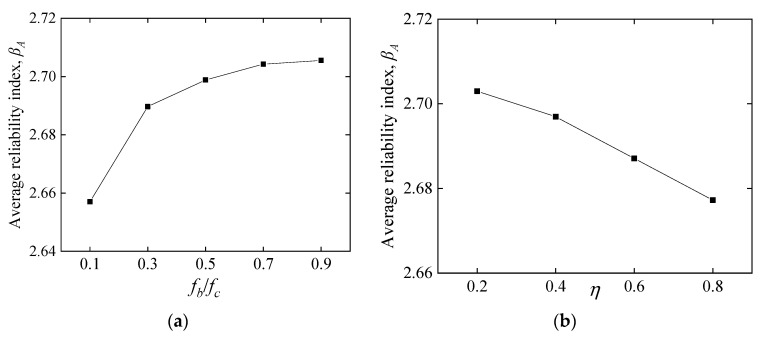

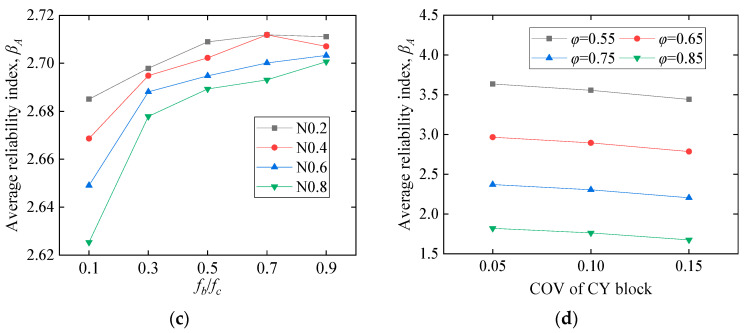
Effect of CY block properties on the average reliability index. (**a**) Effect of *f_b_/f_c_* on *β_A,_* (**b**) effect of *η* on *β_A_*, (**c**) the combined effect of *f_b_/f_c_* and *η* on *β_A_*, (**d**) effect of cov of CY block on *β_A_*.

**Figure 11:**
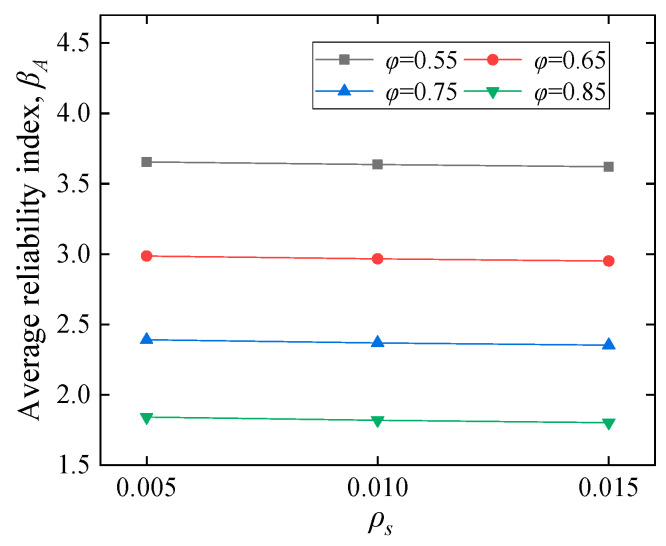
Steel reinforcement ratio effects.

**Figure 12:**
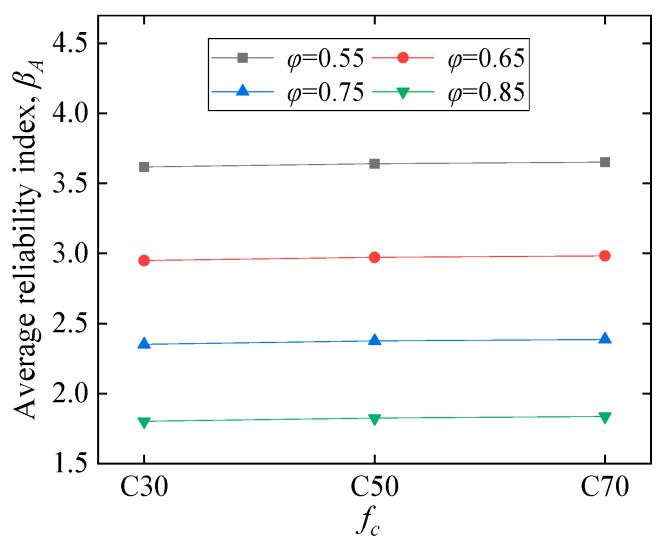
Concrete strength effects.

**Figure 13:**
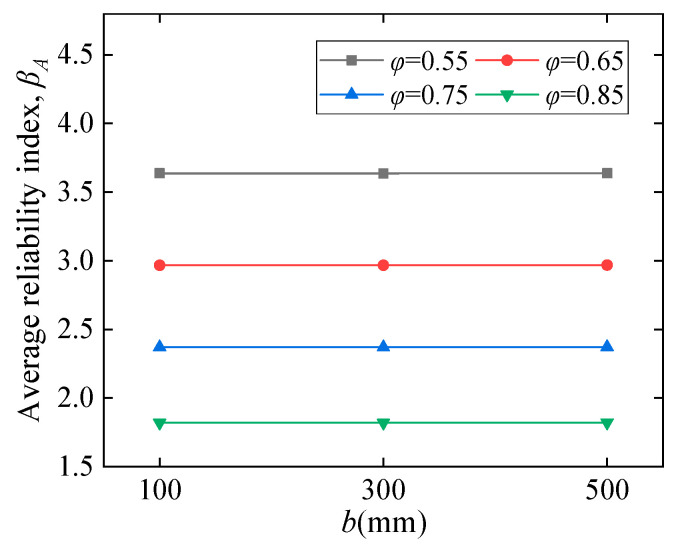
Geometrical dimension effects.

Figure 10 shows the influence of CY block characteristics on the average reliability index *β_A_*. Figure 10a illustrates that *β_A_* first increases suddenly followed by a slower trend as the *f_b_* increases from 0.1 to 0.9 *f_c_*. In Figure 10b, *β_A_* decreases almost linearly with increasing *η*. The label ‘N’ in Figure 10c refers to the embedding depth ratio of the CY block to the beam height, e.g., N0.2 represents a CY block embedding depth of 0.2*d*. Figure 10c demonstrates the combined effect of *f_b_* and *η* on *β_A_*, which is discussed separately in Figure 10a,b. The results indicate that *β_A_* decreases as *η* increases, and it still has a variation trend similar to Figure 10a. The results demonstrated in Figure 10d show that *β_A_* decreases obviously with increasing COV. As the CY block is a material that can be designed, COV will be further reduced in the continued development of CY materials. The *ρ_s_*, *f_c_*, and *b* have not shown apparent influence on *β_A_*, as displayed in Figure 11, Figure 12, and Figure 13, respectively. It can be suggested that the higher of CY block strength with smaller embedding depth *η,* and the lower COV of the CY block is beneficial for *β_A_*.

#### 5.2.2. Influence of Model Uncertainty

**Figure 14:**
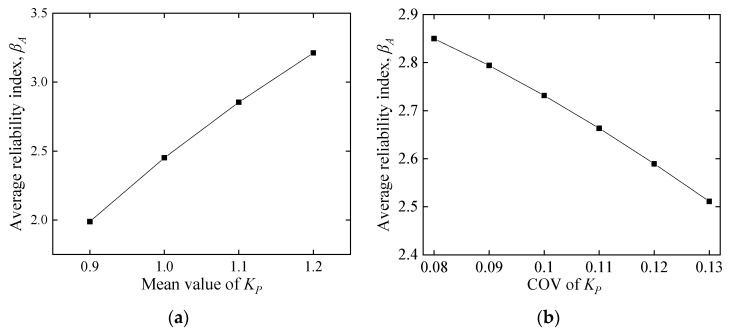
Effect of the model uncertainty factor on the average reliability index. (**a**) Effect of mean value of *K_p_* on *β_A_*, (**b**) effect of COV of *K_p_* on *β_A_*.

As discussed in Section 3.1, for the model uncertainty factor *K_p_*, the mean value and COV are estimated as 1.06 and 0.11, respectively. In this section, the effect of the *K_p_* random features on the reliability index is examined. As depicted in Figure 14, the average reliability index *β_A_* increases dramatically with increasing mean value and decreasing COV, indicating that the random characteristics of *K_p_* are susceptible factors affecting *β_A_*. Hence, it is necessary to collect *K_p_* of CY beams for a scientific assessment and design. However, since the CY beam is currently a relatively new structure, although the current beam-related experiments have proved the effectiveness of the CY mechanism [14,16], it is not enough to evaluate *K_p_*. Further experimental studies are underway to achieve more data. This study focuses on comparing reliability assessments for two structural systems (i.e., FRP-RC beams and CY beams). Thus, it is legitimate to adopt the same *K_p_*.

#### 5.2.3. Influence of the Reduction Factor and Load Effect Ratio

**Figure 15:**
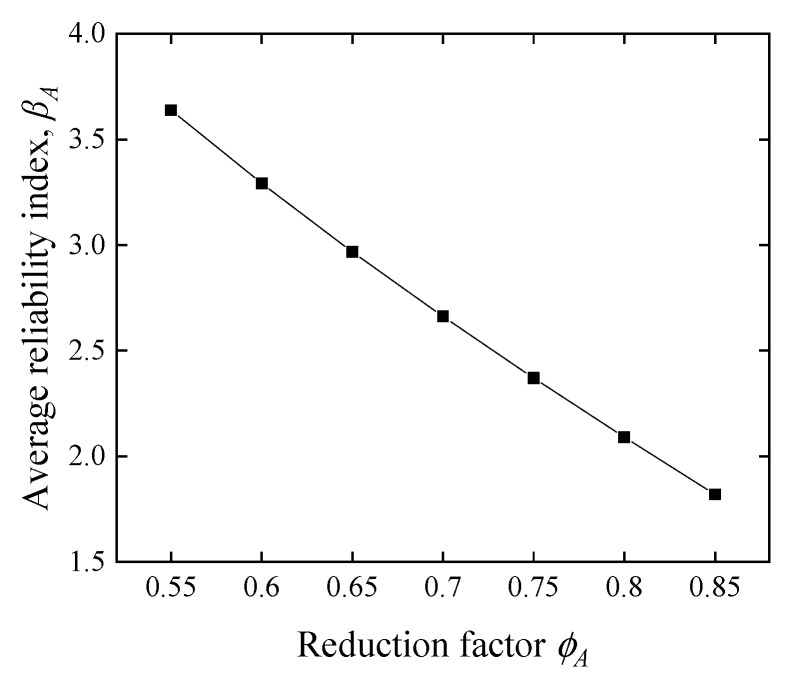
Effect of the reduction factor on the average reliability index.

**Figure 16:**
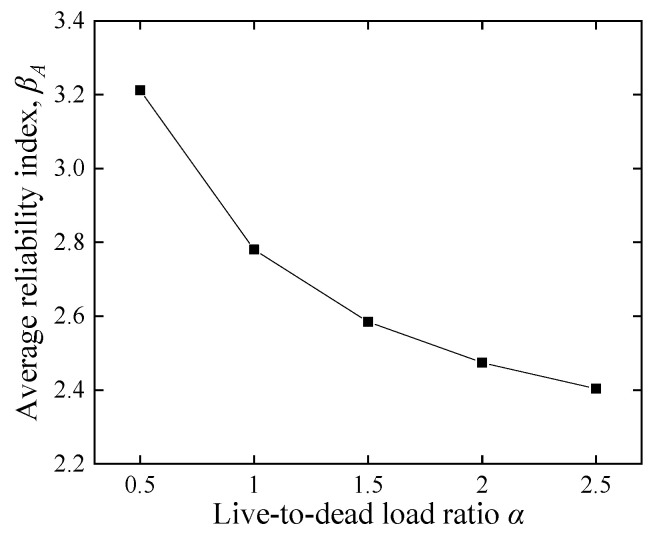
Effect of the live-to-dead load ratio on the average reliability index.

Figure 15 and Figure 16 show that the reduction factor *ϕ_A_* and the live-to-dead load ratio *α* are significant factors for the average reliability index *β_A_*. This trend is consistent with other reliability analyses on FRP-RC beams [49,50]. Figure 15 shows that *β_A_* decreases linearly and dramatically with an increase in the *ϕ_A_*. Since *ϕ_A_* increased from 0.55 to 0.85, *β_A_* dropped from 3.6 to 1.8. Moreover, as the *α* increases, the proportion of live load increases, *β_A_* decreases in a nonlinear curve, and the reduction rate slows down as the load ratio increases. This fact explains that the COV of live loads is higher than that of dead loads (see Table 3), and the distribution type for dead loads is a normal distribution, while live loads are simulated by Extreme type I, with a longer upper tail in probability density.

LRFD, an ideal design method, ensures that a structure or structural component reaches the reliability index. The failure of brittle structures is unwarned, and a smaller reduction factor is required to meet a larger reliability index. In contrast, for ductile structures, the requirement of target reliability is relatively lower. Therefore, from a cost point of view, the CY beam has a higher utilization ratio of the loading capacity than the FRP-RC beam, and it is more economical than FRP-RC beams.

#### 5.2.4. Influence of the Post-Yield Modulus Ratio

**Figure 17:**
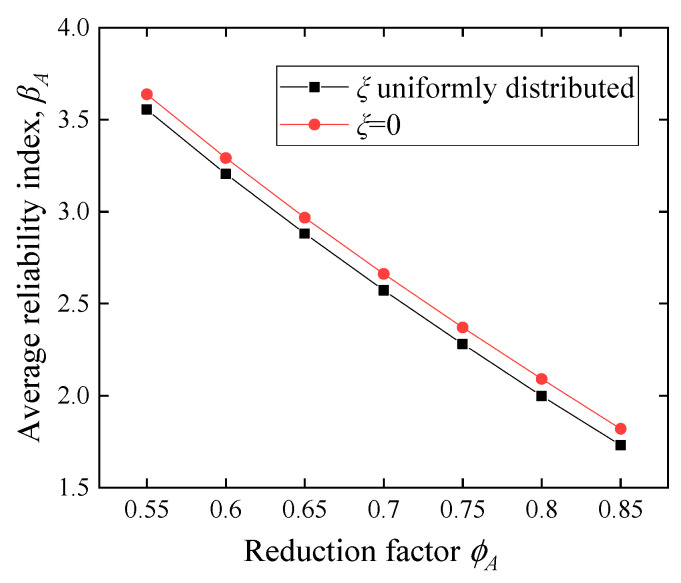
Effect of *ξ* on the average reliability index.

The maximum moment *M_m_* (Equation (19)) is related to the value of the post-yield modulus *ξ* [20]. In the analysis of the above sections, *ξ* = 0. The range of *ξ* has been defined previously and changes with the cross-sectional parameters. To explore the influence of *ξ* on reliability, the analysis of the changes in *ξ* in this section is listed separately. This section assumes that *ξ* is a uniformly distributed variable between *ξ_max_* and *ξ_min_*, and the uncertainties of the other parameters are shown in Table 3 and Table 4. As presented in Figure 17, it is evident that when *ξ* is a variable, *β_A_* is approximately 0.1 lower than *ξ* = 0 under the same reduction factor. The explanation for this phenomenon is that when *ξ* fluctuates, many values fall in the region less than 0, which reduces the *M_m_* value.

Theoretically, for a fixed value of *f_b_*, as *E_b_* decreases, i.e., the *ε_by_* increases, the case gradually increases from I to III (Figure 5). However, due to design variables’ uncertainties, the possible condition of the beam section may be different from that foreseen at the design stage. When *ξ* is a uniformly distributed variable, the probability of these disparities is presented in Table 5. If *E_b_* ≥ 12,500 MPa, it is observed that the cases will not change and all results in I. Furthermore, when *ξ* = 0, although there are uncertainties in other variables, the results of the design are consistent with the calculation. Notably, Case Ⅰ is desirable, and the CY block does not fully yield when the FRP reaches *ε_fm_* in Cases II and III. Therefore, for the CY block, smaller fluctuations of *ξ* (approximately 0) and greater *E_b_* values are beneficial.

## 6. Reliability Analysis Considering the Replacement of the CY Block

### 6.1. Failure Probability of the CY Beam

There are two defined failure modes for CY beams (Figure 3) [20]: (1) failure mode I—the CY block reaching the ultimate strain *ε_bu_*; and (2) failure mode II—the moment resistance drops below the allowable limit *δ_d_* before CY block failure. The failure mode II can be avoided at the design stage. For failure mode I, it will not happen by replacing the CY block before reaching the *ε_bu_*. Event A is defined as the load effect exceeding point A in Figure 3, and event B is defined as the CY block having reached *ε_bu_* but without a timely replacement. The probability of occurrence for two events is *P_A_* and *P_B_*, respectively, and the corresponding LSF is listed in Equations (34) and (43), respectively. Then, the complete failure probability of the structure is *P_s_* = *P_A_* × *P_B_*. Notably, the evidence for *P_s_* expression is that although A and B are not independent events when point A is reached, point B may not be reached under displacement control. Whether the CY block can be replaced at the right opportunity affects the *P_B_*, which is related to the subsequent maintenance strategy. Only when both A and B occur does the CY beams unable to recover their bearing capacity, which is the actual failure of the CY structure. Section 5 discusses the reliability analysis of event *P_A_*, and the following section gives the analysis method and results of *P_B_*.

### 6.2. Reliability Analysis Based on Deformation Control

The CY beam will show a visible ductile deformation before the CY block reaches the ultimate strain *ε_bu_*, so that it is possible to consider the replacement of the CY block at a certain deformation of the beam. Assuming that the curvature and strain relationship is linear, the LSF *Z_B_* can be established based on the curvature (Equation (43)).

(43) $$Z_{B}=\kappa_{u}-\kappa_{re}$$

with

(44) $$\kappa_{re}=\phi_{B}\kappa_{u}$$

where *κ_u_* is the curvature when the CY block reaches ultimate strain *ε_bu_*, which was deduced in [20]:

(45) $$\kappa_{u}=\frac{C_{2}+\sqrt{C_{2}^{2}+4C_{1}C_{3}}}{2C_{1}}$$

where

(46) $$C_{1}=E_{f}\rho_{f}d+\xi E_{b}\eta^{2}d/2;\;C_{2}=E_{f}\rho_{f}\varepsilon_{cu}+E_{f}\varepsilon_{by}\eta +\left(\varepsilon_{cu}-\varepsilon_{by}\right)E_{b}\xi \eta +E_{s}\varepsilon_{sy}\rho_{s};\;C_{3}=\frac{f_{c}\varepsilon_{cu}}{2d}$$

*κ_re_* is a derived random variable representing the curvature of the replacement CY block point. It is a function of several random variables, which can be designed by the reduction factor *ϕ_B_*. In Equation (43), *Z_B_* determines the state of the CY beam with respect to deformations. 

The case of *Z_B_* > 0 represents successful CY block replacement at the design point, and *Z_B_* < 0 indicates a failure to replace.

**Figure 18:**
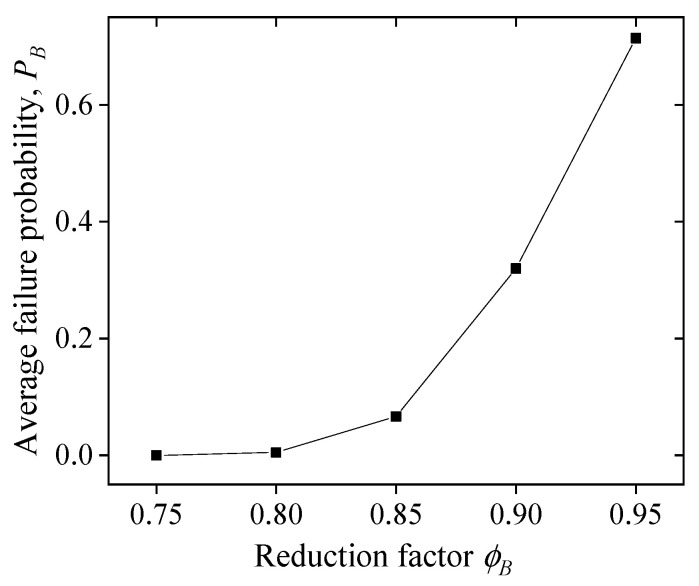
Effect of the reduction factor on the average failure probability, *P_B_*.

The same process of the reliability analysis is adopted as Figure 8, and the material parameters are in keeping with those in Section 5.2. Figure 18 shows the average failure probability *P_B_* versus the reduction factor *ϕ_B_*. The results illustrate that the *P_B_* first increases slightly as *ϕ_B_* increases from 0.75 to 0.85 and then increases significantly as *ϕ_B_* increases from 0.85 to 0.95.

### 6.3. Structural Failure Probability

**Figure 19:**
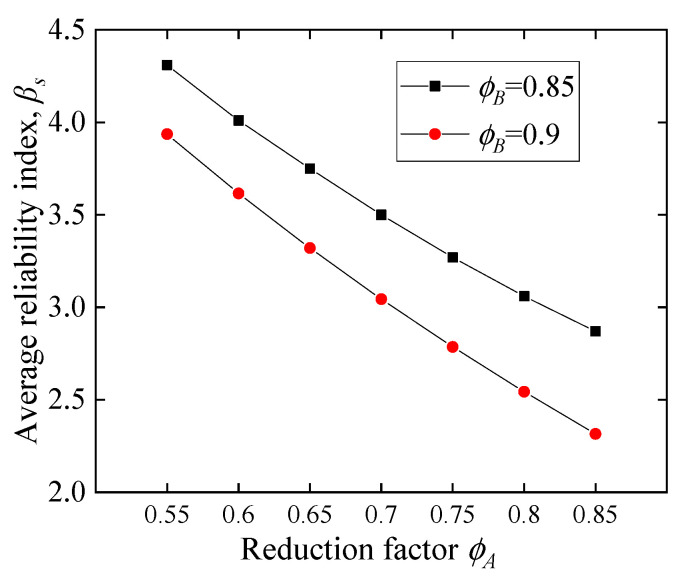
The average reliability index with different reduction factors.

The failure probability *P_s_* and average reliability index *β_s_* of the CY beam are shown in both Figure 19 and Table 6, where *β_A_* adopts the results in Section 5.2.3 and two examples of *P_B_* are taken in this section: *ϕ_B_ =* 0.85 and 0.9, the corresponding *P_B_* is 0.06 and 0.3. Table 6 shows that by considering the replacement of the CY block, the failure probability of the structure is significantly reduced, e.g., when *ϕ_A_* = 0.7, considering the replacement of the CY block with *ϕ_B_* = 0.9, the probability of failure decreases from 3.89 × 10^−3^ to 1.17 × 10^−3^. Changing *ϕ_B_* from 0.9 to 0.85 can achieve a higher reliability index, e.g., the probability of failure decreases from 1.17 × 10^−3^ to 4.50 × 10^−4^ when *ϕ_A_* = 0.7. The relation between the target reliability index and the reduction factor is a curtail for the economic design of the CY beam, and a detailed discussion is given in the following section.

In GB50068-2018 [4], *β_T_* under the ultimate limit state of the 50-year reference period is determined by comprehensively considering safety measures and failure modes, as concluded in Table 7. For FRP-RC beams, the reduction factor recommended by ACI 440 is 0.65 or 0.55 to achieve the *β_T_* of 3.5–4.0 [6]. As a brittle structure, the FRP-RC beam cannot be designed for Class Ⅰ since the required level is 4.2. For the CY beam, *β_T_* of ductile structures for Class Ⅰ is 3.7. For this target, the combination of *ϕ_A_* = 0.55–0.6 and *ϕ_B_* = 0.9 is available (Table 6). It is particularly worth noting that it can be adjusted *ϕ_B_* to meet *β_T_*. For example, the combination of *ϕ_A_* = 0.65 and *ϕ_B_* = 0.85 can also achieve *β_T_*, which is 3.75 in Table 6.

In summary, the *β_T_* for the CY beam is lower than that of the FRP-RC beam, and the utilization of structural bearing capacity results from coordination between the initial safety margin and subsequent maintenance. Particularly, since CY beams have no steel corrosion problems during their life cycle, the loss of bearing capacity is limited, and the failure consequences of CY beams are not serious, so CY beams should have a lower *β_T_* than traditional ductile structures.

## 7. Conclusions

This study presents a reliability-based design analysis of CY beams. Structural failure is defined as the occurrence of both strength and deformation limits. The reliability index or failure probability is determined by the importance sampling (IS) method. Focusing on the limit state of peak flexural bearing capacity, the effect of the design variables on the reliability index is discussed, and recommendations for CY beams in the design process are provided. For the limit state of deformation, the structural failure probability can be achieved based on a combination of the two limit state. Based on the reliability analysis of the CY beam, the following comparison can be drawn:

(1) The characteristics of the CY block are the main reasons affecting the reliability based on ultimate flexural strength (*β_A_*), particularly for the composition of a CY block with higher strength and smaller depth that can improve *β_A_*.
(2) For the parameters that require more experimental results to determine, i.e., COV of CY block strength *f_b_*, model uncertainty *K_p_*, and COV of yield modulus ratio *ξ*, the result indicates that a smaller COV of *f_b_*, a larger mean value and smaller COV of *K_p_*, and a smaller the fluctuation of *ξ* will improve *β_A_*. In addition, a small elastic modulus *E_b_* should be avoided to keep the design and calculation case consistent at the peak point of the bearing capacity.
(3) The effects of the concrete strength, reinforcement ratio, and geometric dimension on the *β_A_* are not significant. The reduction factor and load ratio show considerable influence on *β_A_*, but this issue is challenging to address in a semi-probabilistic design.
(4) The target reliability *β_T_* of the CY beam is referred to as a ductile structure in the guidelines of this study. Compared with the over-designed FRP-RC beam, the safety factor of the CY beam is lower.

The above work provides reference suggestions for the design of the CY beam and establishes future research objectives: (1) a smaller COV and higher *E_b_* of the CY block should be realized to achieve higher reliability and ideal cases; (2) the statistical parameters of *K_p_* of the CY beam and *ξ* of the CY block need to be further determined by accumulated experimental work or civil engineering construction; and (3) the *β_T_* for the CY beam is different from the general ductile structure and needs to be reconstructed.

## Figures and Tables

**Table 1:** Statistical table of the uncertainty factor *K_P_*.

Mean	Std	COV ^1^	Number	Source
0.96	0.175	0.182	3	[22]
0.977	0.051	0.052	6	[23]
0.884	0.088	0.100	6	[24]
0.916	0.12	0.131	6	[25]
0.824	0.039	0.047	3	[25]
1.104	0.037	0.033	2	[26]
1.127	0.122	0.108	4	[27]
1.106	0.076	0.069	5	[28]
1.060	0.068	0.064	4	[29]
0.936	0.115	0.123	4	[30]
1.090	0.101	0.093	6	[31]
1.175	0.099	0.085	9	[32]
0.956	0.128	0.134	5	[33]
1.168	0.108	0.093	3	[34]
1.058	0.041	0.039	5	[35]
1.292	0.041	0.032	4	[36]
1.074	0.101	0.094	6	[37]
1.249	0.073	0.058	4	[38]
1.215	0.134	0.110	6	[39]

^1^ Coefficient of variation (COV) = standard deviation (std)/mean value.

**Table 2:** The K-S test result of the model uncertainty.

Probabilistic Distribution	Test Result	*p* Value	Mean	Std
Normal	0	0.11	1.06	0.12
Lognormal	1	0.05	1.06	0.02
Weibull	1	0.09	1.06	0.02
Extreme type Ⅰ	1	0.00	1.06	0.02

**Table 3:** Probabilistic characteristics of resistance design variables and loads.

Variable			Bias ^1^	COV	Distribution	Source
Geometry		Width *b*	1.01	0.04	Normal	[40]
		Height *h*	1.01	0.04	Normal	[40]
Materials	Concrete	Compressive strength *f_c_*	1.15	0.1	Lognormal	[41]
	Steel	Area *A_s_*	1	0.03	Normal	[42]
		Steel yield strength *f_y_*	1.1	0.075	Normal	[43]
	CY block	CY block yield strength *f_b_*	1.1	0.1	Normal	[17]
Loads		Dead *D_n_*	1.05	0.1	Normal	[44]
		Live *L_n_*	1	0.25	Extreme type I	[44]

^1^ Bias = mean value/standard value.

**Table 4:** Summary of design variables.

Design Variables		Probabilistic Distribution		Values	
Geometry		Width *b* (mm)	100	300	500
		Depth *d* (mm)		2b	
Materials	Concrete	Compressive strength *f_c_* (MPa)	30 50 70		
	Steel	Area *A_s_* (mm^2^)	0.5%:0.5%:1.5%		
		Steel yield strength *f_y_* (MPa)		335	
	CY Block	CY Block yield strength *f_b_* (MPa)	0.1*f_c_*:0.2*f_c_*:0.9*f_c_*		
		Ratio of the height of the CY block to the depth of the beam *η*	0.2:0.2:0.8		
Loads		Live-to-dead load ratio *α*	0.5:0.5:2.5		

**Table 5:** The probability of calculation case.

Design Case	Calculation Case	Probability
Ⅰ	Ⅰ	0.98
	Ⅱ	0.03
	Ⅲ	--
Ⅱ	Ⅰ	--
	Ⅱ	0.96
	Ⅲ	0.04
Ⅲ	Ⅰ	--
	Ⅱ	0.50
	Ⅲ	0.50

**Table 6:** Reliability and failure probability of CY beam under different reduction factors.

Failure Probability		*ϕ_A_* = 0.55	*ϕ_A_* = 0.6	*ϕ_A_* = 0.65	*ϕ_A_* = 0.7	*ϕ_A_* = 0.75	*ϕ_A_* = 0.8	*ϕ_A_* = 0.85
*β_A_*		3.64	3.29	2.97	2.66	2.37	2.09	1.82
*P_A_*		1.38 × 10^−4^	4.99 × 10^−4^	1.50 × 10^−3^	3.89 × 10^−3^	8.89 × 10^−3^	1.83 × 10^−2^	3.44 × 10^−2^
*P_s_*	*ϕ_B_* = 0.9	4.14 × 10^−5^	1.50 × 10^−4^	4.50 × 10^−4^	1.17 × 10^−3^	2.67 × 10^−3^	5.49 × 10^−3^	1.03 × 10^−2^
	*ϕ_B_* = 0.85	8.28 × 10^−6^	2.99 × 10^−5^	9.00 × 10^−5^	2.33 × 10^−4^	5.33 × 10^−4^	1.10 × 10^−3^	2.06 × 10^−3^
*β_s_*	*ϕ_B_* = 0.9	3.94	3.62	3.32	3.04	2.79	2.54	2.31
	*ϕ_B_* = 0.85	4.31	4.01	3.75	3.50	3.27	3.06	2.87

**Table 7:** Target reliability index *β_T_* at the ultimate state of structures under the 50-year period [4].

Structural Class	Consequences of Failure	Target Reliability Index *β_T_*	
		Ductile Failure	Brittle Failure
Class I	High	3.7	4.2
Class II	Medium	3.2	3.7
Class III	Low	2.7	3.2

## Data Availability

All data, models, or code generated or used during the study are available from the corresponding author by request.

## Nomenclature

CY beam				
*b*	breadth of the beam	*δ_d_*		allowable rate of the moment drop
*d*	effective depth of the beam	*γ_D_*		partial safety factor for the dead load
*E_b_*	elastic modulus of CY block equal to *f_b_/ε_by_*	*γ_L_*		partial safety factor for the live load
*E_c_*	elastic modulus of concrete	*η*		ratio of the height of the CY block to depth of the beam
*E_f_*	elastic modulus of FRP	*κ_m_*		curvature at the attainment of the maximum FRP strain
*E_s_*	elastic modulus of the steel bar	*κ_emi_*		equivalent curvature
*f_b_*	yield strength of the CY block	*κ_u_*		curvature at ultimate failure
*f_bu_*	ultimate strength of the CY block	*κ_y_*		yield curvature of the CY section
*f_c_*	compressive strength of concrete	*ρ_f_*		FRP reinforcement ratio
*M_d_*	design load-carrying capacity	*α*		the nominal live-to-dead load ratio
*M_m_*	the moment at attainment of the maximum FRP strain	*ρ_s_*		compression steel reinforcement ratio
*M_n_*	the nominal value of *M_m_*	*ξ*		post-yield modulus ratio
*M_u_*	moment at ultimate failure	*ξ_max_*		maximum value of *ξ*
*S_d_*	the design load	*ξ_min_*	minimum value of *ξ*	
*K_p_*	the model uncertainty factor	*ζ*	distance from the centroid of the steel reinforcement to the top fiber of the beam divided by d	
*ε_by_*	yielding strain of the CY block			
*ε_bu_*	ultimate strain of the CY block	ACI beam					
*ε_cmi_*	strain at concrete–CY block interface at attainment of the maximum FRP strain
		*f_f_*		the tensile stress of the FRP reinforcement
*ε_cu_*	crushing/ultimate strain of concrete	*f_fu_*		the design tensile strength of FRP
*ε_fm_*	maximum allowable strain in FRP	*M_n_*		the nominal bending resistance
*ε_fu_*	ultimate strain of FRP	*M_f_*		the factored bending moment calculated from factored loads.
*ε_sy_*	yield strain of the steel reinforcement	*ϕ*		the strength-reduction factor
*ε*_0_	concrete strain at peak stress	*β*_1_		the equivalent stress block parameter (parameter associated with the depth of the stress block)
*ϕ_A_*	the strength-reduction factor of the CY beam			
*ϕ_B_*	the deformation-reduction factor of the CY beam	*ρ_fb_*		the actual value of the balanced FRP ratio (ACI)
